# Medical College Association

**Published:** 1896-06

**Authors:** 


### Medical College Association.

The Association of American Medical Colleges held its annual meeting in Atlanta, May 4th. The business transacted was formal and routine. The requirements for membership remain the same, namely: Preliminary examinations in 1, English: 2, mathematics; 3, physics; 4, Latin and four years of study in a medical college, at least six months each year. Dr. James M. Bodine, of Louisville, Ky., was elected president, and Dr. Bayard Holmes, of Chicago, re-elected secretary. The work of the Committee on Syllabus was accepted and the committee continued.

